# Improved Multiple-Model Adaptive Estimation Method for Integrated Navigation with Time-Varying Noise

**DOI:** 10.3390/s22165976

**Published:** 2022-08-10

**Authors:** Jinhao Song, Jie Li, Xiaokai Wei, Chenjun Hu, Zeyu Zhang, Lening Zhao, Yubing Jiao

**Affiliations:** 1 National Key Laboratory for Electronic Measurement Technology, North University of China, Taiyuan 030051, China; 2 Aerospace System Engineering Shanghai, Shanghai 201108, China

**Keywords:** multiple-model adaptive estimation, Sage–Husa, adaptive Kalman filter

## Abstract

The accurate noise parameter is essential for the Kalman filter to obtain optimal estimates. However, problems such as variations in the noise environment and measurement anomalies can cause degradation of estimation accuracy or even divergence. The adaptive Kalman filter can simultaneously estimate state and noise parameters, while its performance will also be degraded in complex noise. To address the problem of estimation accuracy degradation and result divergence of the integrated navigation system in a complex time-varying noise environment, an improved multiple-model adaptive estimation (MMAE) that combines the Sage–Husa adaptive unscented Kalman filter with the MMAE is proposed in this paper. The forgetting factor is included as an unknown parameter of MMAE so that the algorithm can adjust the value of the forgetting factor according to different system states. In addition, we improve the hypothesis testing algorithm of classical MMAE to deal with the competition problem of undesirable models that severely impacts the performance of variable-parameter MMAE and enhance the algorithm’s parameter identification capability. Simulation results show that this method enhances the system’s robustness to noises of different statistical properties and improves the estimation accuracy of the filter in time-varying noise environments.

## 1. Introduction

The strapdown inertial navigation system (SINS) is a navigation method in which the inertial sensors (gyroscope and accelerometer) are fixed directly to the body. In contrast to platform inertial navigation, which requires a complex mechanical and physical platform structure, the SINS adopts an algorithmic platform to solve for attitude, velocity, and position information of the body, which has the advantages of small size, light weight, strong overload resistance, simple hardware structure, and comprehensive output information, and is widely used for attitude measurement and navigation in missiles, vehicles, and aerospace [1,2,3].

Nevertheless, inertial navigation systems have the issue of high short-term accuracy and severe long-term drift in practice. It is hard to rely on their information for correction, which dramatically affects the accuracy of inertial navigation [4]. Therefore, other navigation systems such as odometers, magnetometers, and global positioning systems (GPSs) are often used to compensate for inertial navigation systems [5].

Moreover, information from different sources often contains some uncertain noise. These noises cannot usually be accurately measured and simply removed, whereas the characteristic statistical parameters can be reckoned from the physical model characteristics of the system. Therefore, algorithms that can obtain helpful information in dynamic systems with uncertain noisy information and perform optimal state estimation based on the prediction of the next moment are incredibly critical [6].

The Kalman filter (KF) is an optimal state estimation algorithm proposed by Kalman in the 1960s, which uses prior knowledge such as deterministic and statistical characteristic parameters of the system and measurement information to obtain the optimal state estimation [7]. The standard Kalman filter can only solve the estimation problem of linear systems. For nonlinear systems, linear approximations can cause significant errors. To solve this problem, many scholars have improved the standard Kalman filter.

The extended Kalman filter (EKF) is used for the state estimation of nonlinear systems. It uses the Taylor series expansion method to linearize the nonlinear model to make it conform to the Kalman filter model [8]. However, under strong nonlinearity, the above defects have not been significantly corrected [9]. Furthermore, the EKF needs to compute the Jacobian matrix; however, in some cases, the Jacobian matrix has no solution.

In 1995, Julier and Uhlmann proposed the unscented Kalman filter (UKF). The UKF, also known as the sigma-point Kalman filter, transforms the n-dimensional state vector and error covariance matrix to the 2n-dimensional parallel state vector by unscented transformation to evaluate the nonlinear propagation of error covariance rather than by linearization [10]. Therefore, the UKF can accurately obtain the second-order posterior mean and covariance for any nonlinear system [11,12].

In theory, the Kalman filter requires an accurate system model and the exact statistical properties of the noise parameters so that the optimal estimate can be obtained. Inaccurate noise parameters can easily lead to divergence of filtering results. To solve this problem, many scholars have performed careful research on the uncertainty of filter noise parameters. Robust Kalman filtering is a method for dealing with estimation problems for uncertain systems [13,14]. Savkin and Petersen proposed a robust state estimation by constructing an ellipsoidal state estimation set of all states consistent with the measured output and the given noise and uncertainty description to determine if the assumed model is consistent with the measurements [14]. Xie and Soh proposed a Riccati equation approach to guarantee the covariance of all acceptable uncertainties is within a certain range [15]. Li et al. presented an improved robust filter with a double state model on the basis of the chi-square distribution of the square of the Mahalanobis distance to adjust the observation noise covariance matrix in INS/GPS integrated navigation, improving the navigation accuracy [16]. In engineering applications, in addition to robust estimation, a more common approach to solving the problem of filter noise parameter uncertainty is adaptive Kalman filtering.

Innovation-based adaptive estimation (IAE) is based on measurement innovation sequences to correct the noise parameter matrix, but the window size predominantly affects the adaptability of the system; choosing the appropriate size of the window is a crucial problem in practice [17,18]. Sun et al. proposed an improved adaptive UKF to estimate the statistical properties of the measurement noise of the navigation system and applied it to INS/BDS integrated navigation. The simulation results showed that the positioning accuracy was improved compared with the EKF and the adaptive UKF [19]. Narasimhappa et al. proposed a modified Sage–Husa adaptive Kalman filter to incorporate a time-varying noise estimator and robustifier. Experimental results show the performance of the proposed method on a three-axis IMU to minimize the random noise and drift error under static and dynamic conditions [20]. In recent years, with the development of artificial intelligence, some scholars have applied AI algorithms such as neural networks and swarm intelligence to adaptive parameter identification, where training the model with previous data enables the algorithm to quickly estimate the current statistical properties of the noise. However, these methods often require a long time to train the model, and the training result is strongly influenced by the system state of the training process [21,22,23].

In 1964, Magill proposed multiple-model adaptive estimation (MMAE), which uses a set of parallel filters to estimate systems containing unknown parameters [24]. The model parameters of classical MMAE are fixed, and there is no interaction between models, which may have performance degradation or excessive convergence due to inaccurate modeling and competition of undesirable models [25,26]. Assa and Plataniotis proposed a new fusion scheme for MMAE using similarity estimation instead of traditional Bayesian estimation as the hypothesis testing algorithm to solve the undesirable competition among models [27]. Jia and Xu et al. analyzed the mechanism of MMAE and proposed various methods to improve MMAE, and the simulation showed that this indeed improved the performance of MMAE [28]. Li et al. adopted the exponential decay terms penalty function to overcome the excessive convergence problem of classical MMAE and apply it to the problem of unknown Martian atmospheric density during Mars probe landing [29]. Interacting multiple-model estimation (IMM) is developed based on MMAE, which introduces interactions between models and modeling jump patterns as Markov chains to improve the computational efficiency of the system [30,31]. Although IMM has improved performance over MMAE, it does not essentially modify the fixed-parameter structure of the multi-model [32]. Variable structure multiple-model (VSMM) is a new multi-model estimation that assumes a varying set of models which adopt a two-layer hierarchical structure [26,33]. Model set adaptation (MSA) identifies the optimal set of models at the current moment based on the system state. The model state estimator is responsible for getting the optimal state estimate in a given model. Where MSA is the critical component of VSMM, how to enhance the intelligence, flexibility, and real-time performance of the VSMM algorithm is the leading research content at present [34,35].

In this paper, we focus on estimation accuracy degradation and result divergence due to improper noise parameters; aiming to improve the robustness of the filter in a complex time-varying noise environment, an improved MMAE with variable parameters model was proposed. The method combines Sage–Husa with MMAE, which greatly reduces the dependence of the multiple-model algorithm on the initial value selection of model parameters. In addition, we found in our simulations that the optimal value of the forgetting factor is not the same for different system states. Although the difference in accuracy among them is not significant, if we can adjust the forgetting factor adaptively at each moment of the algorithm operation, it will significantly improve the overall accuracy. Therefore, we incorporated the forgetting factor into the unknown parameters of the multi-model structure. In addition, the undesirable competition problem of classical MMAE can have a more severe impact on the variable-parameter model, as we detailedly analyze in Section 3.2. We improve the hypothesis testing algorithm of MMAE to reduce the influence of undesirable models on state estimation, and the algorithm’s parameter identification capability is enhanced. The experiments show that the performance of the proposed method is superior to other algorithms in time-varying noise environments, reflecting the effectiveness and superiority of the method.

The rest of this paper is structured as follows. Section 2 presents the formula derivation of the standard UKF and introduces the algorithm of classical MMAE. Section 3 presents the problems of classical MMAE and the details of the proposed method in this paper. Section 4 introduces the design of the simulation experiment, and simulation results are discussed in detail. In Section 5, the conclusions and suggestions regarding future research are presented.

## 2. Mathematical Preliminaries

### 2.1. Unscented Kalman Filter

In order to better deal with nonlinear systems, the UKF was introduced in this paper for state estimation. The UKF uses the unscented transformation to generate sigma points. The mean and variance of the sigma points are the estimated value of the state vector and the error covariance matrix, respectively, which utilizes an estimation method rather than an approximate linearized approximation and therefore has higher accuracy. The mathematical formulation of the UKF is depicted below.

The process and measurement model of nonlinear discrete-time system can be described as follows:

(1) $$\{\begin{array}{l} X_{k}=f(X_{k-1})+\Gamma_{k/k-1}W_{k-1}\\ Z_{k}=h(X_{k})+V_{k} \end{array}$$

where $X_{k}$ is the n-dimensional state vector, $Z_{k}$ is the m-dimensional measurement vector, f(⋅) represents the nonlinear state transition function, h(⋅) represents the nonlinear measurement function, and $\Gamma_{k/k-1}$ is the system noise distribution matrix. $W_{k-1}$ is the system noise vector, and $V_{k}$ is the measurement noise vector; these are both uncorrelated zero-mean Gaussian white noises. The expectation values of system noise and measurement noise can be expressed as:

(2) $$\begin{array}{l} E\left[W_{k}\right]=0\\ E\left[V_{k}\right]=0\\ E\left[W_{k}W_{j}^{T}\right]=Q_{k}\delta_{kj}\\ E\left[V_{k}V_{j}^{T}\right]=R_{k}\delta_{kj}\\ E\left[W_{k}V_{j}^{T}\right]=0 \end{array}$$

where $Q_{k}$ is system noise covariance, $R_{k}$ is measure noise covariance, and $\delta_{kj}$ is defined as follows:

(3) $$\delta_{kj}=\{\begin{array}{l} 1,\;\mathrm{if}\;k=j\\ 0,\;\mathrm{otherwise} \end{array}$$

The standard UKF algorithm can be described as follows:

Step 1: Initialization

(4) $$\{\begin{array}{l} {\hat{X}}_{0}=E\;[X_{0}]\\ P_{0}=E\;[\left(X_{0}-{\hat{X}}_{0}\right){\left(X_{0}-{\hat{X}}_{0}\right)}^{T}] \end{array}$$

where P is the estimation error covariance.

Step 2: Sigma-point calculation

(5) $$\{\begin{array}{l} \xi_{0}=\bar{X}\\ \xi_{i}=\bar{X}+{\left(\sqrt{\left(n+\kappa \right)P_{x}}\right)}_{i},\;i=1,\;2,\;\ldots \;,\;n\\ \xi_{i+n}=\bar{X}-{\left(\sqrt{\left(n+\kappa \right)P_{x}}\right)}_{i} \end{array}$$

(6) $$W_{i}^{m}=\{\begin{array}{ll} \lambda /\left(n+\lambda \right), & i=0\\ 1/2\left(n+\lambda \right), & i\neq 0 \end{array}$$

(7) $$W_{i}^{c}=\{\begin{array}{ll} \lambda /\left(n+\lambda \right)+1+\beta -a^{2}, & i=0\\ 1/2\left(n+\lambda \right), & i\neq 0 \end{array}$$

(8) $$\lambda =a^{2}\left(n+\kappa \right)-n$$

where $W_{i}^{m}$ and $W_{i}^{c}$ are weights, n is the state dimension, κ and a are tuning parameters, and λ is the composite parameter.

Step 3: State prediction

Following the sigma sampling strategy in Step 2, sigma points $\xi_{i}$ are computed from ${\bar{X}}_{k-1}$ and $P_{k-1}$, and $\gamma^{i}{}_{k/k-1}$ is obtained by propagating the nonlinear state function f(⋅), evaluating the one-step state estimation ${\hat{X}}_{k/k-1}^{}$ and the error covariance matrix $P_{k/k-1}$.

(9) $$\gamma^{i}{}_{k/k-1}=f\left(\xi^{i}{}_{k-1}\right),\;i=0,\;1,\ldots ,\;2n$$

(10) $$\{\begin{array}{l} {\hat{X}}_{k}^{}={\hat{X}}_{k/k-1}^{}+K_{k}\left(Z_{k}-{\hat{Z}}_{k/k-1}\right)\\ K_{k}=P_{XZ}P_{ZZ}^{-1}\\ P_{k}=P_{k/k-1}-K_{k}P_{ZZ}K_{k}^{T} \end{array}$$

(11) $$P_{k/k-1}=\sum_{i=0}^{2n}W_{i}^{c}\left(\gamma_{k/k-1}^{i}-{\hat{X}}_{k/k-1}^{}\right){\left(\gamma_{k/k-1}^{i}-{\hat{X}}_{k/k-1}^{}\right)}^{T}+Q_{k-1}$$

Step 4: Measurement update

Similarly, the sigma point is calculated using ${\hat{X}}_{k/k-1}^{}$ and $P_{k/k-1}$ according to Step 2, and using the nonlinear measurement function h(⋅) to calculate the $\chi_{k/k-1}^{i}$, the measurement predictions ${\hat{Z}}_{k/k-1}^{}$, autocovariance matrix $P_{ZZ}$, and cross-covariance matrix $P_{XZ}$ are further derived.

(12) $$\chi^{i}{}_{k/k-1}=h\left(\xi^{i}{}_{k/k-1}\right),\;i=0,\;1,\ldots ,\;2n$$

(13) $${\hat{Z}}_{k/k-1}^{}=\sum_{i=0}^{2n}W_{i}^{m}\chi_{k/k-1}^{i}=\sum_{i=0}^{2n}W_{i}^{m}h\left(\xi^{i}{}_{k/k-1}\right)$$

(14) $$P_{ZZ}=\sum_{i=0}^{2n}W_{i}^{c}\left(\chi_{k/k-1}^{i}-{\hat{Z}}_{k/k-1}^{}\right){\left(\chi_{k/k-1}^{i}-{\hat{Z}}_{k/k-1}^{}\right)}^{T}+R_{k-1}$$

(15) $$P_{XZ}=\sum_{i=0}^{2n}W_{i}^{c}\left(\xi^{i}{}_{k-1}-{\hat{X}}_{k/k-1}^{}\right){\left(\chi_{k/k-1}^{i}-{\hat{Z}}_{k/k-1}^{}\right)}^{T}$$

Step 5: Filtering update

(16) $$\{\begin{array}{l} {\hat{X}}_{k}^{}={\hat{X}}_{k/k-1}^{}+K_{k}\left(Z_{k}-{\hat{Z}}_{k/k-1}\right)\\ K_{k}=P_{XZ}P_{ZZ}^{-1}\\ P_{k}=P_{k/k-1}-K_{k}P_{ZZ}K_{k}^{T} \end{array}$$

where $K_{k}$ is the filter gain matrix.

### 2.2. Classical MMAE

MMAE uses a set of parallel filters to estimate independently, and each model corresponds to different unknown parameters. The model’s probability is calculated according to the hypothesis test algorithm, and the final state output is obtained by weighted summation of each model state estimation. MMAE enhances the system’s adaptability to noise by increasing the number of models with different unknown parameters. This approach increases the computational effort; however it is stable and generally does not suffer from divergent estimation results.

The classical MMAE is given as follows.

(17) $$p_{k,i}=\beta e^{\left[-\frac{1}{2}\delta {\tilde{Z}}_{k,i}^{T}S_{k,i}^{-1}\delta {\tilde{Z}}^{}{}_{k,i}\right]}$$

where $S_{k,i}=H_{k,i}P_{k/k-1,i}H_{k,i}^{T}+R_{k,i}$, $\beta =\frac{1}{\sqrt{{\left(2\pi \right)}^{m}\left|S_{k,i}\right|}}$, m is the dimension of vector, $p_{k,i}$ represents the probability of the ith model, ${\tilde{Z}}_{k,i}$ is the measurement innovation, i represents the ith model, and k is the simulation step.

The weight of each model can be formulated as follow:

(18) $$W_{k,i}=\frac{W_{k-1,i}\cdot p_{k,i}}{\sum_{i=1}^{n}W_{k-1,i}\cdot p_{k,i}}$$

The final state estimation is obtained by weighted summation of each model:

(19) $${\hat{X}}_{MMAE|k}=\sum_{i=1}^{n}{\hat{X}}_{k,i}W_{k,i}$$

## 3. Proposed Method

In general, MMAE assigns a suitable posterior conditional probability for each model to ensure that the final estimation converges to a proper range. From the perspective of probability theory, it belongs to an optimal estimate. However, after a lot of simulation verification, we find that the classical MMAE has the following two inherent problems in practice.

1. The classical multi-model algorithm suffers from the issue of undesirable competition of unnecessary models [26]. Specifically, model probabilities are assigned based on innovation; however, models with large innovations are likewise assigned a certain probability. In addition, during weight updating, the model weights of the previous moment need to be considered besides the probability that each model is assigned at the current moment. With the combined effect of these two factors, some poor models are sometimes assigned certain weights, and undesirable competition emerges. Especially in time-varying noise environments, when the noise environment changes, the trend is difficult to reverse even though the converged model is no longer the optimal current model [27,36].
2. The selection of model parameters is usually in a bounded range based on analysis of system characteristics and the significance of parameters, where fixed parameters are selected to constitute the model [37]. In theory, the actual model must be in this range. However, the noise characteristics of measurement anomalies and composite application scenarios are difficult to predict and accurately model. For example, the harsh road conditions of vehicle navigation, the interference and obscuration of GPS signals, the vibration difference of coal cutters under various working conditions, and noise characteristics also change, and such variations are often complex and cannot be accurately predicted and modeled. On the one hand, too few models may fail to cover the actual model, making the MMAE algorithm lose its advantage. On the other hand, too many models will lead to a manifold increase in computational effort and undesirable competition of unnecessary models [26]. Therefore, for MMAE with fixed parameters, selecting an appropriate model set is particularly crucial but not easy to achieve.

Therefore, in order to solve the problem of estimation accuracy degradation caused by improper noise parameter setting and limited coverage of the classical MMAE model, aiming to enhance the adaptability of the system to time-varying noise and the ability to deal with nonlinear systems, we proposed an improved structure of the MMAE, which combined Sage–Husa with MMAE. Specifically, we replace the standard Kalman filter of the classical MMAE with the Sage–Husa adaptive UKF. This variable-parameter multi-model structure reduces the system’s dependence on the selection of initial noise parameters and extends the coverage of the MMAE model.

### 3.1. Sage–Husa Adaptive UKF

The UKF has excellent advantages for dealing with nonlinear systems; however, the model divergence problem of the UKF is also more severe when the noise parameters are inaccurate [38]. To better respond to complex noise variations and exert the advantage of the UKF. We adopt Sage–Husa adaptive UKF as the model instead of the standard Kalman filter like the classical MMAE. The Sage–Husa adaptive filter can estimate the noise parameters online according to the measurement innovation in real-time, avoiding the problem of estimation accuracy degradation caused by the unreasonable noise parameter matrix setting [38,39]. Referring to the mathematical derivation of the UKF given in the previous part, the Sage–Husa adaptive UKF is shown as follows.

In the filter, measurement prediction error (measurement innovation) is:

(20) $$\delta {\tilde{Z}}_{k/k-1}=Z_{k}-{\hat{Z}}_{k/k-1}\approx H_{k/k-1}{\tilde{X}}_{k/k-1}+V_{k}$$

where $H_{k/k-1}={\frac{\delta h}{\delta X}|}_{{\hat{X}}_{k/k-1}}$.

The Sage–Husa covariance estimator is given as:

(21) $$\begin{array}{l} {\hat{R}}_{k}=\left(1-d_{k}\right){\hat{R}}_{k-1}+d_{k}\rho_{k}\\ {\hat{Q}}_{k}=\left(1-d_{k}\right){\hat{Q}}_{k-1}+d_{k}\sigma_{k} \end{array}$$

(22) $$\begin{array}{l} \rho_{k}=\delta {\tilde{Z}}_{k/k-1}\delta {\tilde{Z}}_{k/k-1}^{T}-H_{k}P_{k/k-1}H_{k}^{T}\\ \sigma_{k}=K_{k}\delta {\tilde{Z}}_{k/k-1}\delta {\tilde{Z}}_{k/k-1}^{T}K_{k}^{T}+P_{k}^{}-\Phi_{k/k-1}P_{k-1}\Phi_{k/k-1}^{T} \end{array}$$

where $H_{k}={\frac{\delta h}{\delta X}|}_{{\hat{X}}_{k}}$, $d_{k}=\frac{1-b}{1-b^{k+1}}$, and $d_{0}=1$, b is the forgetting factor.

In the standard KF, the gain calculation loop where the noise parameter matrix is located is relatively independent of the state estimation loop. In contrast, the noise parameter matrix in the Sage–Husa adaptive filter is influenced by the measurement value, thus turning the system into a complex nonlinear system that is more severely affected by the estimation anomalies. In addition, according to Equation (22), we find that measurement innovation simultaneously acts on the parameter estimation process of Q and R, and both Q and R have an effect on the state estimation of the filter. Therefore, if the Sage–Husa adaptive filter is used to estimate Q and R simultaneously, it would be easy to diverge the filtering results. Fortunately, in integrated navigation systems, the measurement noise is more susceptible to the complex noise environment, while the variation of progress noise is relatively small. Therefore, in this work, we use Sage–Husa to estimate the measurement noise in real-time and incorporate the process noise as a fixed unknown parameter into the multi-model structure. Doing so ensures the adaptability of the filter and enhances the stability of the system.

### 3.2. Improved Hypothesis Testing Algorithm

Classical MMAE can be affected by undesirable models with undesirable competition due to the inherent characteristics of the multiple-model algorithm. For applications where the noise parameters variation is slight, the undesirable competition only affects the convergence rate of the model without having a severe impact on the estimation accuracy. However, for environments where the noise parameters can vary, undesirable competition can seriously affect the weight assignment of the optimal model, which extends detection time and reduces the system’s decision-making capability, especially when the optimal model has changed and the innovation differences between models are delicate.

When performing further analysis (Equation (17)), we noticed that MMAE assigns weights mainly based on the innovation of each model. Thus, $-\frac{1}{2}\delta {\tilde{Z}}_{k,i}^{T}S_{k,i}\delta {\tilde{Z}}_{k/k-1}$ plays a decisive role in weight assignment. The constant term ($\frac{1}{2}$) is called the penalty value. The penalty value also has an impact on the probability assignment of the model. Therefore, we will explore the relationship between penalty value and weight assignment through mathematical derivation.

Firstly, we define two models to perform state estimation simultaneously and at the k moment, according to Equation (17), The calculation results of the two models are as follows:

(23) $$\begin{array}{l} \phi_{a}=\delta {\tilde{Z}}_{k,a}^{T}S_{k,a}^{-1}\delta {\tilde{Z}}^{}{}_{k,a}\\ \phi_{b}=\delta {\tilde{Z}}_{T}^{k,b}S_{k,b}^{-1}\delta {\tilde{Z}}^{}{}_{k,b} \end{array}$$

where a and b represent the model a and model b, respectively, and suppose $\phi_{a}<\phi_{b}$.

Then the weight of model a being assigned at this moment is:

(24) $$\begin{array}{ll} W_{k,a} & =\frac{W_{k-1,a}\cdot p_{k,a}}{W_{k-1,a}\cdot p_{k,a}+W_{k-1,b}\cdot p_{k,b}}\\ & =\frac{W_{k-1,a}\cdot e^{-n\cdot \phi_{a}}}{W_{k-1,a}\cdot e^{-n\cdot \phi_{a}}+W_{k-1,b}\cdot e^{-n\cdot \phi_{b}}} \end{array}$$

where n is the penalty value.

Derivative of Equation (24) with respect to p:

(25) $$\frac{\partial W_{k,a}}{\partial n}=\frac{W_{k-1,a}W_{k-1,b}\left(\phi_{b}-\phi_{a}\right)e^{n\cdot \phi_{a}}e^{n\cdot \phi_{b}}}{{\left(W_{k-1,a}\cdot e^{n\cdot \phi_{a}}+W_{k-1,b}\cdot e^{n\cdot \phi_{b}}\right)}^{2}}$$

It can be seen from Equation (25) that the derivative is always greater than zero; therefore, the weight of model a being assigned increases as the value of the forgetting factor increases.

Through analysis, we can acquire that if we enlarge the penalty value, in the same circumstances, the model that is closest to the real model is assigned a greater weight. In other words, the larger the penalty value, the optimal model will be assigned a more significant weight more quickly. However, probability jumps may occur, resulting in a decrease in estimation accuracy, especially in the early stages of the algorithm operation, in order to maintain stability during the early stages when the model parameters vary considerably, as well as to still possess the ability to adjust the model weights and alleviate the problem of undesirable competition after a period of time. Inspired by [29], which introduced an exponentially decaying penalty value function to alleviate the over-convergence problem, we introduce an increasing function that replaced the constant penalty value to deal with the contradiction between convergence speed and stability, enhancing the model identification capability of the system. In our simulation experiment, the penalty value function was set as:

(26) $$\rho \left(k\right)=0.5\cdot k^{0.15}$$

In Equation (17), we found that in addition to the exponential term, there is also an $s_{k}$ term containing the internal parameters of the filter in the β term, leading to a greater probability that the model with the smaller β is assigned among the two filters with similar innovation, which is apparently contrary to our expectations that the exponential term plays a decisive role in the probability calculation [28]. What is worse, for variable-parameter models, the differences in noise parameters between models become small after a period of operation, which causes the harm of this problem to be more severe, and decrease the algorithm’s decision-making capability. Therefore, we removed the β term. Since the weights of the models are determined by the proportion of each model to the sum of all models, removing the β term will have no effect on the algorithm’s operation. Then the hypothesis testing algorithm can be expressed as:

(27) $$\Lambda_{k,i}=e^{\left[-\rho \left(k\right)\delta {\tilde{Z}}_{k,i}^{T}S_{k,i}^{-1}\delta {\tilde{Z}}^{}{}_{k,i}\right]}$$

### 3.3. Improved MMAE

As mentioned above, we combined the Sage–Husa adaptive UKF with MMAE to fully exploit their advantage. The forgetting factor has an effect on the estimation results, which determines the confidence of the adaptive system for prior information. The smaller the forgetting factor is set, the stronger the adaptability of the system; however, when the estimated parameters differ from the actual parameters by a small amount, if the forgetting factor is set small, it may lead to jumps in the parameters and reduce the stability of the estimation, thus affecting the filtering accuracy. In general, the forgetting factor needs to select the optimal value for the current moment to ensure a balance between system adaptability and stability; however, it is hard to achieve in practice. Therefore, in addition to the process noise matrix, the forgetting factor is also included in the unknown parameters of the MMAE, which enables the algorithm to identify the optimal forgetting factor value among the artificially set parameters, reducing the system’s dependence on the selection of the forgetting factor. Moreover, the undesirable competition problem can seriously affect the system’s ability to respond to complex time-varying noise. In this regard, we propose MMAE with an improved hypothesis testing algorithm to overcome the problem.

**Figure 1:**
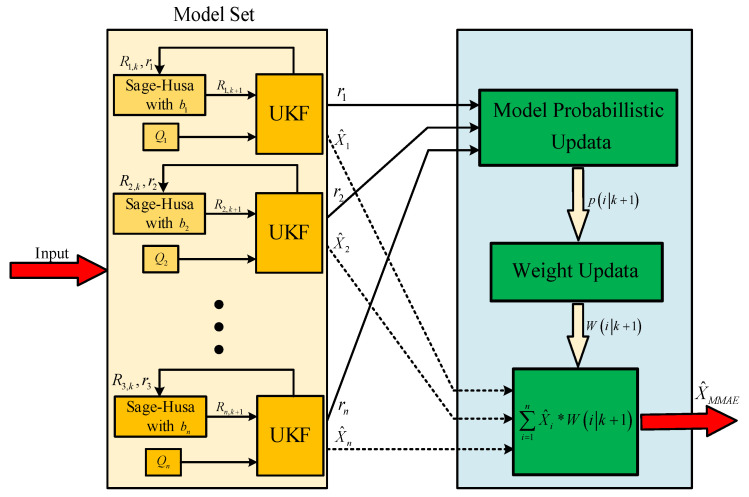
The structure diagram of improved MMAE.

The specific process of improved MMAE implementation is as follows, and the structure diagram is shown in Figure 1.

Step 1: Set up m filters and assign different forgetting factors and noise parameter matrices to them.

Step 2: Initialize each model to a weight of 1/m.

Step 3: Each filter performs state estimation independently and calculates the measurement innovations.

Step 4: The model corrects the measurement noise matrix according to the innovation and the corresponding forgetting factor using the Sage–Husa algorithm.

Step 5: Update the filter weights based on the measurement innovations using an improved hypothesis testing algorithm.

Step 6: Weighted summation of the estimated vectors for each filter to obtain the final state output.

Step 7: Update the data and repeat Steps 3–6.

## 4. Experiment and Result Analysis

**Figure 2:**
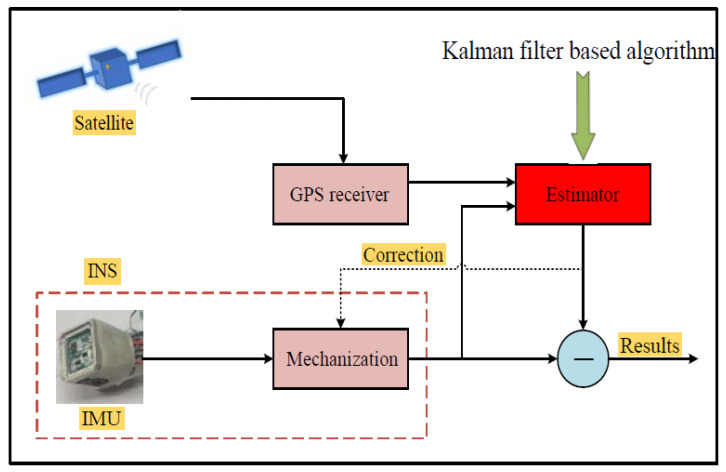
The structure of INS/GPS integrated navigation.

GPS positioning error does not drift with time and can maintain high accuracy in long-time navigation, which complements INS and becomes the first choice for inertial-based integrated navigation. The INS/GPS integrated navigation structure is shown in Figure 2.

**Figure 3:**
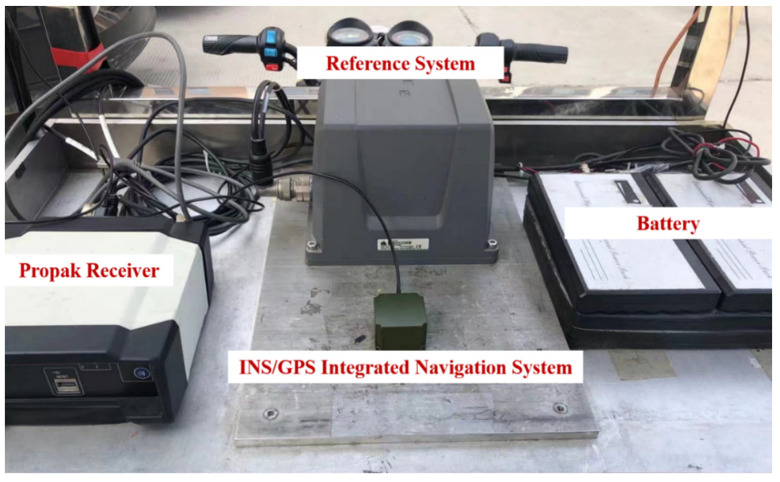
Vehicle navigation platform.

In this section, we applied the algorithm to INS/GPS vehicle integrated navigation and carried out three experiments. Experiment 1 is used to verify the effect of the forgetting factor on estimation accuracy under different system states. Experiment 2 is designed to test the effectiveness of the improved hypothesis testing algorithm of MMAE for mitigating competition of undesirable models. Experiment 3 by comparing the positioning accuracy of different algorithms to test the effectiveness and meliority of the proposed method. A vehicle navigation platform, as shown in Figure 3, is used to collect INS and GPS navigation data. The INS is composed of three sets of MEMS accelerometers and gyroscopes; NEO-M8T was used as the GPS receiver. A high-precision navigation device was installed on the vehicle navigation platform to collect accurate navigation data as the reference. These instruments have been carefully calibrated and compensated for the lever arm error. The parameters of the above navigation device are shown in Table 1.

In addition, we used the mean squared error (MSE) of position error to quantificationally evaluate the performance of algorithms. MSE is the mean square of the distance between the predicted and actual values, reflecting the extent of data deviating from actual values and is inversely proportional to navigation accuracy. MSE can be defined as follows:

(28) $$\mathrm{MSE}=\frac{1}{n}\sum_{i=1}^{n}{\left(Y_{i}-{\hat{Y}}_{i}\right)}^{2}$$

Experiment 1:

In this paper, we include the forgetting factor as an unknown parameter of MMAE to improve the performance of the algorithm in time-varying noise. That is because after $d_{k}$ convergence, models with smaller forgetting factors will have higher adaptivity. They can better respond to changes in noise, while models with larger forgetting factors will demonstrate better stability when the noise parameter matrix is almost constant. To verify this, we carried out an experiment in which the integrated navigation data were simulated with three different values of forgetting factors. In order to simulate the process of noise parameters changing after $d_{k}$ convergence, we used forgetting factor values instead of $d_{k}$ for the experiment. Three time periods were selected as observation intervals, and the MSE was calculated for the northward error within the interval, and the results are shown in Table 2.

In 0–100 s, the adaptive system estimates the measurement noise parameters, and the filter with a smaller forgetting factor has more adaptability, achieving a better performance. While the estimation accuracy of the filter with large values of the forgetting factor is higher when the variation of the noise parameter matrix tends to stabilize. That is consistent with our previous analysis. Although the difference in accuracy is not significant, if we can select the optimal forgetting factor for each moment based on the system state while the algorithm is running, there will be a substantial improvement in the overall accuracy, especially for systems in complex noise environments.

**Figure 4:**
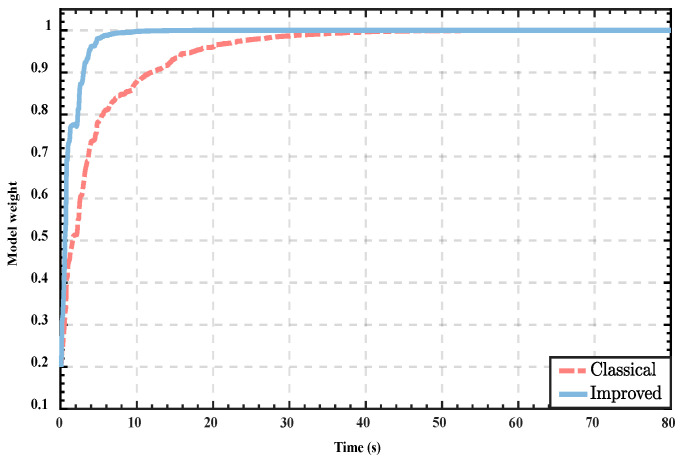
Weight change of the optimal model.

Experiment 2:

For variable-parameter models, undesirable competition can affect the convergence rate of the optimal model and extend detection time, especially when the parameters of other models do not differ much from the optimal model; the optimal model could even not be assigned the maximum weights due to the influence of previous data. Therefore, we improve the hypothesis testing algorithm of the classical MMAE. To verify the effectiveness of the improved method in alleviating the undesirable competition problem, we use a set of INS/GPS navigation data and perform the combined navigation solution with the traditional MMAE and the improved method, respectively. The weight change process of the optimal model was recorded and shown in Figure 4.

According to the result of Figure 4, it can be noticed that the optimal model of the classical MMAE suffers from the competition from undesirable models, and the convergence rate of the optimal model is slow. In contrast, the convergence rate of the improved algorithm is significantly greater than the classical algorithm. From the results, the improved method significantly alleviates the undesirable competition and reduces the algorithm’s identification time, which can dramatically improve the performance of the variable-parameter MMAE in the time-varying noise environment.

Experiment 3:

In this part, we used a set of INS/GPS vehicle navigation data collected by the vehicle navigation platform, as shown in Figure 3, to evaluate the effectiveness of the proposed method. Furthermore, the meliority of the proposed method was evaluated by comparing the results with the standard UKF, classical MMAE, and Sage–Husa UKF. In addition, we replace the penalty value function in our method with the exponentially decaying penalty value function proposed in [29] to verify the superiority of our improvement. The vehicle navigation platform simulates regular vehicle driving at the test site and records navigation data for 3500 s. In order to simulate the time-varying noise environment and evaluate the adaptability of the algorithm to complex noise, we artificially added position noise to the measurement data; specific parameters are shown in Table 3.

**Figure 5:**
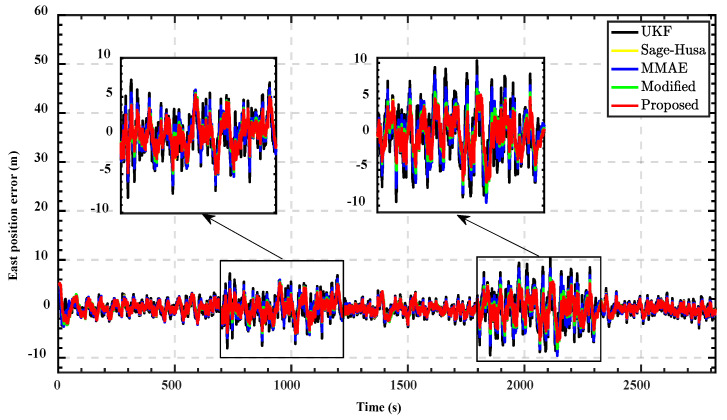
East position errors comparison of different method.

**Figure 6:**
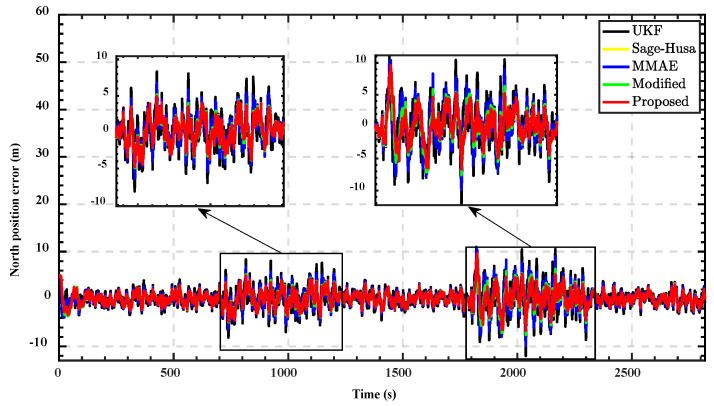
North position errors comparison of different method.

Subsequently, we used different integrated navigation algorithms to solve the data and compare the results with the navigation results of the reference device to obtain the position errors. The position errors in the east direction for different algorithms are demonstrated in Figure 5, and the north position errors are shown in Figure 6. In order to quantitatively evaluate the performance of different algorithms, the MSE of the position error in three observation intervals are shown in Table 4.

From the results, the standard UKF cannot respond well to changes in noise characteristics due to it having no adaptability. Its performance is the worst among the five algorithms during these three time periods. MMAE uses a set of parallel filters to estimate the state vectors simultaneously, each filter corresponding to a different noise parameter; the results of each filter are weighted and summed to derive the final state estimate. It expands the coverage of the noise parameter model to a certain extent, and the estimation accuracy is improved compared with the standard UKF. However, models with fixed parameters and a finite number can hardly cover the whole characteristics of varying noise. Therefore, there is only a limited improvement in accuracy.

The adaptability of Sage–Husa adaptive UKF for changing noise is significantly higher than that of the standard UKF and classical MMAE. During the three observation periods, the accuracy is improved apparently. That is because it can estimate noise parameters in real-time while performing parameter estimation, which enables the system to follow the variation of the noise environment well. However, a single forgetting factor and process noise parameter matrix cannot guarantee that Sage–Husa gets the optimal estimation at every moment, which dramatically limits the performance.

The exponential decay penalty value function proposed in [29] enables the weights of each model to converge to an appropriate range. However, the recognition speed of the algorithm decreases with decreasing penalty values, which affects the performance of the algorithm in time-varying noise.

The improved MMAE takes advantage of Sage–Husa adaptive estimation and MMAE. The forgetting factor and the process noise parameter matrix are set as unknown parameters of the multi-model structure. The improved probability assignment function significantly alleviates the undesirable competition problem and enhances the ability of the algorithm to assign model weights. This makes it feasible for the proposed method to select the optimal value of the forgetting factor according to different system states. The stability and adaptability of the system are simultaneously guaranteed. The performance is better than the other four algorithms under the varying noise environment.

**Figure 7:**
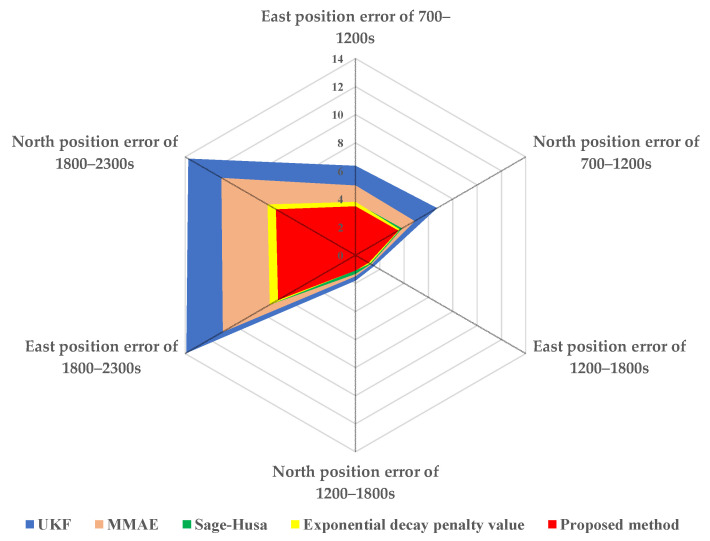
Radar map for MSE of position error of four methods.

In order to show the performance difference between the five algorithms more intuitively, the radar chart for MSE of position error as shown in Table 4 is demonstrated in Figure 7. It can be seen from Figure 7 that the performance of the proposed method is significantly superior to other methods.

## 5. Conclusions

In this work, an improved multiple-model adaptive estimation method was developed, taking advantage of MMAE and Sage–Husa. The performance of the proposed method in integrated navigation simulation is higher than the UKF, Sage–Husa, and classical MMAE. Furthermore, it is easy to extend the algorithm to pattern recognition, target tracking, and fault detection to reduce system dependence on noise parameter selection and improve filter robustness to time-varying noise. In addition, how to enhance filter stability when measurement anomalies and reduce adaptive system complexity will be our subject in the future.

## Figures and Tables

**Table 1:** The parameters of navigation devices.

Device	Parameter	Value
INS	Gyro bias	$0.5^{{}^{\circ}}/h$
	Gyro random walk	$0.2^{{}^{\circ}}/\sqrt{h}$
	Accelerometer bias	10 mg
GPS	Velocity precision	0.5 m/s
	Position precision	3 m
Reference device	Velocity precision	0.03 m/s
	Position precision	1 cm

**Table 2:** MSE of position errors of different forgetting factors.

Forgetting Factor (b)	0–100 s	200–300 s	400–500 s
0.95	3.4178	2.2838	2.7963
0.97	3.5405	2.1956	2.7265
0.99	3.6641	2.1116	2.6564

**Table 3:** Noise parameters.

	700 s–1200 s (m)	1800 s–2300 s (m)
East Position	6 × randn	9 × randn
North Position	6 × randn	9 × randn

Where randn is a random number subject to normal distribution.

**Table 4:** MSE of position errors of different methods.

Error (m)	Algorithm	700 s–1200 s	1200 s–1800 s	1800 s–2300 s
East position error (m)	Standard UKF	6.3643	1.5321	13.8936
	Sage–Husa	3.7254	1.2423	6.9714
	MMAE	4.9558	1.3075	10.8799
	Exponential decay	3.7961	1.1767	7.0322
	Proposed method	3.4774	1.0694	6.3463
North position error (m)	Standard UKF	6.6866	1.8067	13.7319
	Sage–Husa	3.8142	1.3226	7.2156
	MMAE	4.8517	1.4829	10.9979
	Exponential decay	3.6485	1.0659	7.2199
	Proposed method	3.5103	1.0846	6.5168

## Data Availability

Not applicable.
